# Particulate matter 10 exposure affects intestinal functionality in both inflamed 2D intestinal epithelial cell and 3D intestinal organoid models

**DOI:** 10.3389/fimmu.2023.1168064

**Published:** 2023-06-26

**Authors:** Ye Seul Son, Naeun Son, Won Dong Yu, Aruem Baek, Young-Jun Park, Moo-Seung Lee, Seon-Jin Lee, Dae-Soo Kim, Mi-Young Son

**Affiliations:** ^1^ Department of Stem Cell Convergence Research Center, Korea Research Institute of Bioscience and Biotechnology (KRIBB), Daejeon, Republic of Korea; ^2^ Department of Bio-Molecular Science, Korea Research Institute of Bioscience and Biotechnology (KRIBB) School of Bioscience, University of Science and Technology (UST), Daejeon, Republic of Korea; ^3^ Environmental Disease Research Center, Korea Research Institute of Bioscience and Biotechnology (KRIBB), Daejeon, Republic of Korea; ^4^ Digital Biotech Innovation Center, Korea Research Institute of Bioscience and Biotechnology (KRIBB), Daejeon, Republic of Korea

**Keywords:** human intestinal epithelium, human intestinal organoid, particulate matter 10, inflammation, calcium signaling, absorptive function

## Abstract

**Background:**

A growing body of evidence suggests that particulate matter (PM10) enters the gastrointestinal (GI) tract directly, causing the GI epithelial cells to function less efficiently, leading to inflammation and an imbalance in the gut microbiome. PM10 may, however, act as an exacerbation factor in patients with inflamed intestinal epithelium, which is associated with inflammatory bowel disease.

**Objective:**

The purpose of this study was to dissect the pathology mechanism of PM10 exposure in inflamed intestines.

**Methods:**

In this study, we established chronically inflamed intestinal epithelium models utilizing two-dimensional (2D) human intestinal epithelial cells (hIECs) and 3D human intestinal organoids (hIOs), which mimic *in vivo* cellular diversity and function, in order to examine the deleterious effects of PM10 in human intestine-like *in vitro* models.

**Results:**

Inflamed 2D hIECs and 3D hIOs exhibited pathological features, such as inflammation, decreased intestinal markers, and defective epithelial barrier function. In addition, we found that PM10 exposure induced a more severe disturbance of peptide uptake in inflamed 2D hIECs and 3D hIOs than in control cells. This was due to the fact that it interferes with calcium signaling, protein digestion, and absorption pathways. The findings demonstrate that PM10-induced epithelial alterations contribute to the exacerbation of inflammatory disorders caused by the intestine.

**Conclusions:**

According to our findings, 2D hIEC and 3D hIO models could be powerful *in vitro* platforms for the evaluation of the causal relationship between PM exposure and abnormal human intestinal functions.

## 1 Introduction

Since the 1990s, air pollution has intensified globally, and continuous exposure to ambient air pollution can affect mortality risks and morbidity outcomes (1). Particulate matter (PM) is among the most significant air pollutants because it contains organic chemicals, metals, acids, and dust particles (2). It is widely recognized as a major environmental contributor to the global disease burden (3). Based on the aerodynamic diameter of the particles, PM can be classified into coarse (PM10) and fine (PM2.5). In most cases, PM2.5 and PM10 are derived from emissions from different sources (4); PM2.5 is produced by the combustion of gasoline and diesel fuel. In contrast, PM10 is mainly produced by the combustion of agricultural waste, forest fires, construction sites, waste burning, industrial sources, wind-blown dust from open lands, and fragments of fungi and bacteria. In daily life, PM10 concentrations are generally higher than those of PM2.5 (5). Exposure to PM10 is known to trigger inflammation in various diseases, including respiratory diseases, cardiovascular diseases, and skin conditions (6–9).

Evidence from epidemiological studies has demonstrated that PM10 can be absorbed through the gastrointestinal (GI) tract either through direct ingestion of contaminated food or via indirect inhalation (10–12). Inhaled PM10 can be filtered out through mucociliary clearance, such as by coughing, or be transported via the mucus from the lower to the upper airway (13, 14). PM10 in the mucus layer can be swallowed and transported to the GI tract which leads to long-term retention and exposure to high concentrations of PM10. PM10 may also be directly ingested through the consumption of contaminated food or water. Western diets contain nearly 10^3^ particles of PM10, and that value can be used to estimate the amount ingested per day by an individual (15). It has been shown that short- and long-term exposure to PM10 is strongly associated with GI diseases such as appendicitis, gastroenteritis, oxidative stress-mediated hyper gut permeability, inflammatory bowel disease (IBD), Crohn’s disease (CD), and an altered gut microbiome (15–20). However, there are very few reports regarding differential pathological responses to PM10 in healthy controls and patients with intestinal inflammation. It has recently been reported that the co-treatment of PM (PM10, PM2.5) and inflammatory cytokines can enhance the secretion of pro-inflammatory cytokines in human primary olfactory mucosal cells (21). In addition, the underlying biological mechanisms of how PM10 exposure affects the intestine are not fully understood, which explains the lack of a relevant and reproducible *in vitro* intestinal model.

Chronic exposure to PM10 of Caco-2 (19) results in inflammatory responses and oxidative stress, followed by increased epithelial permeability. However, most of the studies were conducted in transformed epithelial cell lines as *in vitro* model systems and *in vivo* mouse models. Although, one recently published study showed that chronic PM exposure decreases the levels of intestinal barrier function-associated proteins in a commercially available epi intestinal tissue model (22). Commonly used *in vitro* intestinal cell models, including Caco-2 and HT29 cell lines derived from human colorectal adenocarcinoma, exhibit unicellular characteristics and altered expression of transporters, failing to recapitulate the functionality of the normal intestinal epithelium (23–25). Therefore, a normal intestine-relevant cell model is necessary for studying the cellular function or molecular mechanism of exposure to environmental toxins, including PM (23).

The necessity of appropriate *in vitro* models has triggered the advancement of intestinal differentiation methods derived from human pluripotent stem cells (hPSCs) (26–28). According to previous studies, hPSC-derived intestinal epithelium models, including 2-dimensional (2D) human intestinal epithelial cells (hIECs) (27) and *in vitro* mature 3D human intestinal organoids (hIOs) induced by interleukin-2 treatment (26) exhibit various intestinal cell types. These include enterocytes, goblet cells, paneth cells, and enteroendocrine cells, as well as cell-type-specific functionality. A variety of transporters can be displayed in these 2D and 3D intestinal epithelial models, including glucose transporters, fructose transporters, and peptide transporters (26, 27).

In this study, we aimed to generate 2D hIEC- and 3D hIO-based chronic inflammation models induced by pro-inflammatory cytokines interferon γ (IFNγ) and tumor necrosis factor α (TNFα), which faithfully recapitulate IBD. Furthermore, we investigated whether the cellular response to PM10 exposure can be influenced by differences in intestinal epithelial status between 2D and 3D normal and chronically inflamed intestinal epithelial cells by dissecting the molecular mechanisms underlying PM10 exposure.

## 2 Materials and methods

### 2.1 Culture and maintenance of hPSC

The human embryonic stem cells (hESCs) line H9 was purchased from WiCell Research Institute (Madison, WI, USA, WA09) and co-cultured with Mitomycin C-treated mouse embryonic fibroblasts (MMC-MEF, AG Scientific, M-1108). hESCs were maintained with hESC medium [Dulbecco’s modified Eagle’s medium (DMEM)/F12 (Gibco/Invitrogen, Carlsbad, CA, USA, 11330057), 1% penicillin/streptomycin (P/S; Gibco, 15140122), 1% GlutaMAX (Gibco, 35050061), 0.1% β-mercaptoethanol (Gibco, 21985023), 10% serum replacement (SR; Gibco, 10828028), and 8 ng/ml basic fibroblast growth factor (R&D Systems, Minneapolis, MN, USA, 4114-TC)]. The medium was replaced daily. Once a week, the cells were dissociated into small fragments and passaged. The hESCs and the animal experiment were approved by the Public Institutional Review Board designated by the Ministry of Health and Welfare (No. P01-201409-ES-01) and the Institutional Animal Care and Use Committee of the Korea Research Institute of Bioscience and Biotechnology (No: KRIBB-AEC-21245), respectively.

### 2.2 hPSC-derived 2D hIECs differentiation

For differentiation of hPSCs into hIECs (27), we initially induced the formation of the definitive endoderm (DE) by treating them with activin A (100 ng/ml; R&D Systems, 338-AC) for three days in RPMI 1640 medium (Gibco, 11875119). The cells were supplemented with 2 mM L-glutamine (Gibco, 25030081), 1% P/S, and increasing concentrations of 0, 0.2, or 2% fetal bovine serum (FBS; Thermo Fisher Scientific Inc. Waltham, MA, USA, 26140) on well-maintained hPSCs with more than 70% confluency. The DE cells were then further differentiated into hindgut (HG) by treatment with fibroblast growth factor 4 (FGF4, 250 ng/ml; Peprotech, Rocky Hill, NJ, USA, 235-F4) and CHIR99021 (1.2 μM; Tocris Bioscience, Minneapolis, MN, USA, 4423) in DMEM/F12 (Gibco) containing 2 mM L-glutamine, 1% P/S, and 2% FBS for four days. Subsequently, the HG cells were dissociated into single-cell suspension using 0.25% trypsin-EDTA (Gibco, 25200072) for 5 min at 37°C, neutralized with DMEM/F12 containing 2% FBS, and harvested. As the cell pellet was centrifuged for 5 min at 1250 rpm, it was resuspended and seeded onto a 1% Matrigel (Corning, NY, USA, 354234)-coated culture plate with hIEC differentiation medium 1 (hIEC medium 1) [DMEM/F12, epithelial growth factor (EGF, 100 ng/ml; R&D Systems, 236-EG), R-spondin1 (100 ng/ml; Peprotech, 120-38), insulin (5 μg/ml; Thermo Fisher Scientific Inc., A11382IJ), 1% N2 supplement (Thermo Fisher Scientific Inc., 17502048), 2 mM l-glutamine, 1% NEAA (Thermo Fisher Scientific Inc., 12587010), and 15 mM HEPES buffer (Thermo Fisher Scientific Inc., 15630080)]. These cells were considered hIEC progenitors, and hIEC medium 1 was replaced daily for seven days. For passaging, hIEC progenitors were dissociated by treatment with trypsin-EDTA for 5 min at 37°C. Following harvesting, hIEC progenitors were neutralized with DMEM/F12 containing 2% FBS and centrifuged for 5 min at 1250 rpm. We then resuspended the cell pellets in hIEC medium 1 and either seeded them onto 1% Matrigel-coated tissue culture plates at a passage ratio of 1:3 or preserved the cells in CryoStor CS10 freezing medium (Stemcell Technologies, Vancouver, Canada, ST07930). To differentiate the hIEC progenitors into functional hIECs, 1.34 x10^5^ cells/cm^2^ hIEC progenitors were passaged onto 1% Matrigel-coated Transwell inserts (Corning, 3460) in hIEC medium 1 containing 10 μM Y-27632 (Tocris, Bristol, UK, 1254). The cells were incubated until they reached near-confluence. Upon confluency, the medium was replated with hIEC differentiation medium (hIEC medium 2) [DMEM/F12, EGF (100ng/ml), Wnt-C59 (2 μM; Selleckchem, Huston, TX, USA, S7037), Valproic acid (VPA, 1 mM; Stemgent, Huston, TX, USA, 04-0007), 2% FBS, 2% B27 supplement, 1% N2 supplement, 2 mM L-glutamine, 1% NEAA, and 15 mM HEPES buffer] to induce differentiation into functional hIECs. Every alternate day, a new medium was used. To generate inflamed 2D hIECs, 10, 50, and 100 ng/ml IFNγ (R&D systems, 285-IF) and 10, 50, and 100 ng/ml TNFα (R&D systems, 210-TA) were differentiated from days 4 to 14. PM10 (Sigma-Aldrich, St. Louis, MO, USA, ERM^®^-CZ100) was administered between days 7 and 14. TEER values were measured using epithelial tissue volt/ohmmeter (EVOM2, WPI, Sarasota, FL, USA) following the manufacturer’s recommendations.

### 2.3 hPSC-derived 3D hIOs differentiation

The 3D hIOs derived from hPSCs were generated as described previously (28). The hPSCs were dissociated into clumps and seeded in Matrigel-coated dishes in mTeSR1 medium (Stemcell Technologies, ST85850). After the hPSCs reached 80–90% confluency, the culture medium was replaced with the DE induction medium [RPMI 1640 (Gibco) containing 1% P/S, 2 mM L-glutamine, 100 ng/ml Activin A (R&D Systems), and 0 – 2% FBS]. After three days, the cells were washed with RPMI 1640 medium, and the medium was replaced with a hindgut induction medium [DMEM/F12 containing 1% P/S, 2 mM L-glutamine, 3 μM CHIR99021, 250 ng/ml FGF4, and 2% FBS]. For a period of 4–6 days, the medium was replaced once every 48 h with fresh medium. Upon creation of the hindgut spheroids, they were collected, washed with advanced DMEM/F-12 (Thermo Fisher Scientific Inc., 12634028), and embedded in a Matrigel dome. The 3D hIOs were cultured in 3D hIOs medium [advanced DMEM/F-12, 1% P/S, 2 mM L-glutamine, 15 mM HEPES, 1× B27, 100 ng/ml EGF, 100 ng/ml noggin (R&D Systems, 6057-NG-01M), and 500 ng/ml R-spondin 1]. Passaging of the 3D hIOs took place every 10–14 days. To generate inflamed 3D hIOs, the 3D hIOs were treated with pro-inflammatory cytokines including 0.5, 1, 5, and 10 ng/ml IFNγ and 2, 20, 40, and 100 ng/ml TNFα after 3–4 days of passaging. PM10 was administered for the final seven days.

### 2.4 RNA preparation, cDNA synthesis, and quantitative real-time PCR

Total RNA was extracted from differentiated cells using a RNeasy Mini Kit (Qiagen, Hilden, Germany, 74106). After extraction, total RNA was reverse-transcribed using a Superscript IV First-Strand Synthesis System kit (Invitrogen, 18091200), in accordance with the manufacturer’s protocol. The PCR was conducted on a 7500 Fast Real-time PCR System (Applied Biosystems, Foster City, CA, USA, 4351107). The target gene expression was normalized to that of the internal control using GAPDH expression. **Supplementary Table 1** lists the primers used.

### 2.5 RNA-sequencing analysis

An Agilent 2100 Bioanalyzer system (Agilent Biotechnologies) was used to analyze RNA samples of high quality with an RNA integrity number (RIN) greater than 7.5. The RNA libraries were prepared according to the manufacturer’s protocols the using Illumina TruSeq library preparation kit (Illumina, San Diego, CA, USA). We performed RNA-seq on an Illumina NextSeq1000 (Illumina) using the standard Illumina RNA-Seq protocol, with a read length of 2 × 100 bases. In order to evaluate the sequence data, the NGSQCToolkit v.2.3.3 was used, and the adapters were removed using Cutadapt v.3.7 with the default settings. Low-quality sequences were trimmed using Sickle v.1.33, with a Phred quality threshold score of 20. A trimmed read that contained ambiguous characters (such as N) or was less than 50 bp, was excluded from the analysis. After preprocessing raw reads, clean reads were mapped to the reference genome (GRCh38) using HISAT2 v.2.2.1, with default parameter settings, and StringTie v.2.2.0 utilizing the reference annotation file to estimate gene and transcript expression levels. Using all protein-coding genes, the multidimensional scaling (MDS) analysis was performed to cluster the samples based on their overall similarity in gene expression patterns. Our objective was to determine whether it was possible to distinguish between phenotype classes based on gene expression patterns. In this analysis, log2(x+1) transformation values were used and rows with zero gene expression were removed from all samples. The MDS analysis of the pairwise distances of the samples was conducted with the help of the functions “cmdscale,” and “dist” (maximum distance measure) in the R v.4.0.2 statistical programming language and plotted using the R package. A Principal Component Analysis (PCA) was computed by eigendecomposing the covariance matrix to identify the principal components. The principal components have been calculated using the “prcomp” function in the R programming language. Spearman’s rank correlation coefficient (Spearman’s correlation) was used to analyze the statistical dependence between the rankings of the two independent samples. Spearman’s correlation was calculated using the “cor” function with ‘spearman’ method in R. In addition, Heatmap and hierarchical clustering were performed using the ‘heatmap.2’ function of the gplots package v.3.1.3 and the Maximum distance by the “hclust” function of the stats package v.3.6.2 in R.

### 2.6 Immunostaining

2D hIECs and 3D hIOs were washed twice with PBS (Thermo Fisher Scientific Inc., 21600010) before being fixed with 4% paraformaldehyde (Sigma-Aldrich, 15710) for 15 min at room temperature. The cells were cryopreserved using the O.C.T. compound (Sakura, USA, HIO-0051) and sectioned at a thickness of 10 μm. After permeabilization with PBS containing 0.1% Triton X-100 (PBS-T; Sigma-Aldrich, T9284) for 15 min, the samples were blocked for 1 h at room temperature with 4% bovine serum albumin solution (BSA; Bovogen Biologicals, Australia, bsa100). In the presence of primary antibodies, the target proteins were captured overnight at 4°C. Slides were washed with PBS-T and incubated for 1 h at room temperature with diluted secondary antibodies at a concentration of 1:200. In addition, the nuclei of stained samples were stained with DAPI and viewed under a fluorescence microscope (Olympus, Japan, IX51) or a confocal microscope (ZEISS, Germany, LSM800). **Supplementary Table 2** lists the antibodies used in this study.

### 2.7 LIVE/DEAD assay

2D hIECs were incubated with the LIVE/DEAD Viability/Cytotoxicity Kit (Invitrogen, L3224) containing calcein-AM (2 μM) and EthD-1 (4 μM) at room temperature for 30 min in order to determine viability and cell death. A fluorescence microscope (Olympus) was used to image cells after they had been washed with PBS. The fluorescence was captured at 494/517 nm and 528/617 nm with calcein-AM and EthD-1, respectively. ImageJ software (NIH, Bethesda, MD, USA) was used to quantify the fluorescence intensity.

### 2.8 Transporter assay

For the measurement of intracellular calcium levels, cells were incubated for 1 h at 37°C with 5 μM Fluo-4 acetoxymethylester (fluo-4 AM, Molecular Probes, Eugene, Oregon, USA, F14201) in a calcium-free isotonic buffer containing 2 mM MgCl_2_ (Sigma-Aldrich, M4880), 5 mM KCl (Sigma-Aldrich, P5405), 5.5 mM D-glucose (Sigma-Aldrich, G7021), 10 mM HEPES, and 140 mM NaCl (Sigma-Aldrich, S7653). Following incubation, the cells were washed with a calcium-free isotonic buffer. The cells were stimulated using a calcium-free isotonic buffer with 10 mM CaCl_2_ (Sigma-Aldrich, C5670) was added for calcium influx, and 50 μM Glu-Phe dipeptide (Sigma-Aldrich, G2752) for stimulation of protein transporter. The fluorescence of cells was monitored with 488 nm excitation, and during recording, the signal was emitted at 505–530 nm. Confocal microscopy and Zen software (ZEISS) were used to image and quantify fluorescence intensity.

### 2.9 Fluorescein isothiocyanate-4kDa dextran assay

For the analysis of intestinal permeability in 2D hIECs, cells were grown on Transwell inserts under each condition. After 14 d, the apical side was treated with 1.25 μM FD4 (Sigma-Aldrich, 46944) in Hank’s balanced salt buffer (HBSS, Gibco, 14025092) for 2 h. The basolateral side was filled only with 1.5 mL HBSS buffer. The basolateral side buffer (100 μL) was analyzed at 485/520 nm using SpectraMax M3 microplate reader (Molecular Devices, Sunnyvale, CA, USA).

### 2.10 Mitochondrial superoxide detection

To detect oxidative stress, the hIECs were cultured under normal and inflamed conditions with or without PM10 medium. Each cell was incubated with 200 nM Mitotracker (Thermo, M7514) and 5 μM MitoSOX™ Red mitochondrial superoxide indicator (Thermo, M36008) in culture medium at 37 °C for 30 min. The cells were captured at a wavelength of 490/516 nm for MitoTracker and 510/580 nm for MitoSOX using a fluorescence microscope (Olympus).

### 2.11 Statistics

Data are displayed as mean ± standard deviation (SD; technical triplicates) or mean ± standard error of the mean (SEM; at least three independent experimental data points). Parametric statistical analyses were conducted using the student’s *t*-test. A statistically significant *p*-value of < 0.05 was determined.

## 3 Results

### 3.1 Establishment of *in vitro* 2D inflamed hIECs

**Figure 1:**
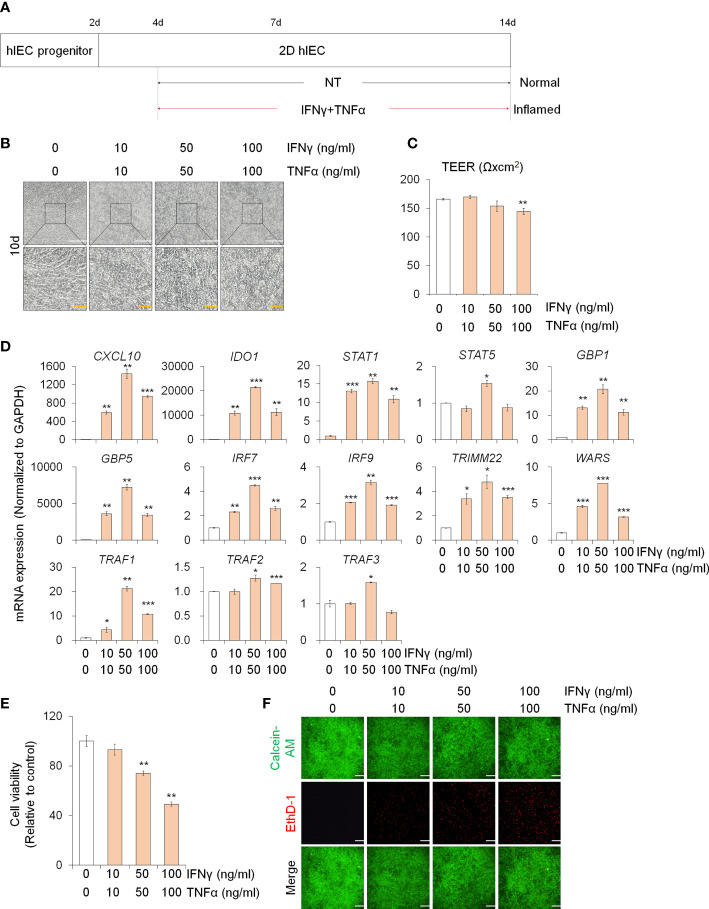
Generation of 2D inflamed hIECs. **(A)** Schemetic diagram of 2D inflamed hIEC generation using IFNγ and TNFα co-treatment. **(B)** Morphological analysis of normal and inflamed hIECs. White scale bar, 200 μm. Yellow scale bar, 50 μm. **(C)** TEER value measurement of normal and inflamed hIECs. Data are means ± SEM (n = 3) **(D)** Relative expression of IFNγ and TNFα response genes after IFNγ and TNFα co-treatment. Data are means ± SD (n = 3). **(E)** Cell viability assay in 2D normal and inflamed hIECs after 0 – 100 ng/ml IFNγ and TNFα treatment, respectively. Data are means ± SD (n = 3). **(F)** Representative images and quantification analysis of LIVE/DEAD assay. Scale bar, 100 μm. Data are means ± SD (n = 3). *P < 0.05, **P < 0.01, and ***P < 0.001 by two-tailed t test.

In order to study the effect of PM10 on inflamed intestinal epithelium, we differentiated hPSCs into 2D hIECs, which are relevant models for the human small intestine (27, 28). The differentiated 2D hIECs contained intestine-specific cells such as intestinal stem cells (*LGR5*), progenitor cells (*CDX2*, *SOX9*), paneth cells (*LYZ*), goblet cells (*MUC2*), endocrine cells (*CHGA*), and enterocytes (*VIL1*) (**Supplementary Figures 1A, B**). To investigate whether PM10 affects sensitivity in normal and inflamed intestinal models, we established an *in vitro* inflamed hIEC model by exposure to IFNγ and TNFα for 10 days (**Figure 1A**). Compared to the cobblestone shape of normal hIECs, co-treatment with IFNγ and TNFα induced an elongated shape in 2D hIECs (**Figure 1B**). Epithelial permeability was significantly decreased in hIECs treated with 100 μg/ml of IFNγ and TNFα (**Figure 1C**). The expression of IFNγ- and TNFα-response genes, such as *CXCL10*, *IDO1*, *STAT1, STAT5, GBP1, GBP5, IRF7*, *IRF9*, *TRIMM22*, *WARS, TRAF1*, *TRAF2*, and *TRAF3*, increased in inflamed hIECs (**Figure 1D**). These results suggest that inflammatory responses were successfully recapitulated in 2D hIECs using IFNγ and TNFα co-treatment. For each cytokine concentration, we chose 50 ng/ml, which represents the observed inflammatory responses without inducing apoptosis (**Figures 1E, F**).

### 3.2 2D inflamed hIECs are more sensitive to PM10 exposure

**Figure 2:**
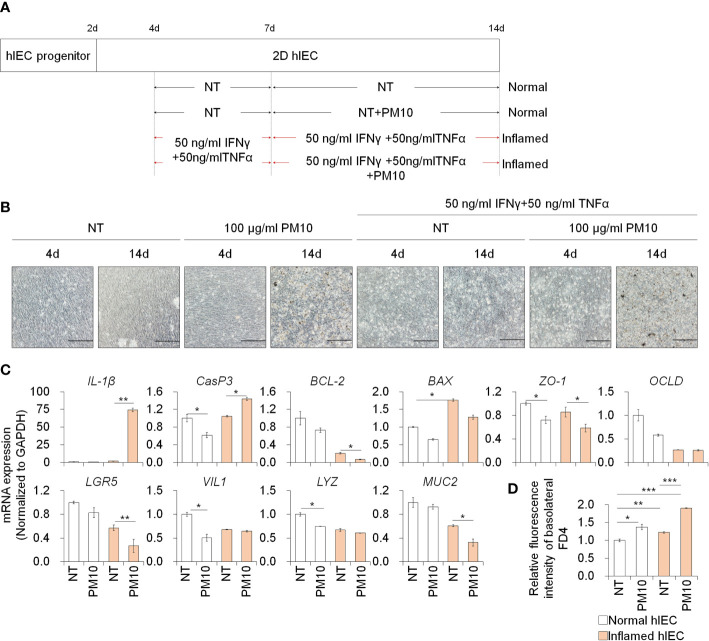
Phenotypic analysis of 2D hIECs following PM10 exposure. **(A)** Schemetic diagram of PM10 treatment in 2D normal and inflamed hIECs induced by 50 ng/ml INFγ and 50 ng/ml TNFα. **(B)** Morphological analysis of normal and inflamed hIECs with or without PM10 treatment (100 μg/ml). Scale bar = 200 μm. **(C)** Relative expression of inflammation, apoptosis, tight junction, and intestinal marker genes in normal and inflamed hIECs with or without PM10 treatment (100 μg/ml). Data are means ± SD (n = 3). *P < 0.05, **P < 0.01, and ***P < 0.001 by two-tailed t test. **(D)** Relative fluorescence intensity of basolateral FD4 in normal and inflamed hIECs with or without PM10 treatment (100 μg/ml). Data are means ± SD (n = 3). *P < 0.05, **P < 0.01, and ***P < 0.001 by one-way ANOVA test.

To assess the effect of PM10 exposure in the inflamed intestinal epithelium, we administered IFNγ and TNFα for 10 days and exposed various concentrations of PM10 in normal and inflamed hIECs for the last 7 days (**Figure 2A**). First, we examined the cytotoxic effect of PM10 (10–1000 μg/mL) on 2D hIECs. In 2D hIECs treated with only 1000 μg/ml of PM10, there was a 50% reduction in viability (**Supplementary Figure 2**, white bars). The cell viability of hIECs was reduced in a dose-dependent manner in response to PM10 inflamed cells (**Supplementary Figure 2**, yellow bars). A concentration range of 100 to 1000 μg/ml of PM10 significantly reduced the viability of inflamed hIECs; therefore, we decided to use 100 µg/ml of PM10 for further experiments. PM10 particles were observed in the cell layer of PM10-treated hIECs (**Figure 2B**). The levels of inflammatory cytokines (*IL-1β*) and apoptosis-related genes (*BAX*) were upregulated in inflamed hIECs compared to those in normal hIECs (**Figure 2C**). Upregulation of *IL-1β*, *Caspase-3* (*CasP3*) expression and downregulation of anti-apoptotic markers (*BCL-2*), intestinal specific markers (*CDX2*, *LGR5*, *VIL1*, *LYZ*, and *MUC2*), and tight junction marker expression were observed in PM10-treated inflamed hIECs (**Figure 2C**). The fluorescence intensities of basolateral FD4 were markedly increased in PM10-treated inflamed hIECs compared to that of normal and inflamed hIECs (**Figure 2D**). These results demonstrate that inflamed intestinal epithelial cells respond more sensitively to PM10 treatment.

### 3.3 PM10 exposure induces calcium signaling dysfunction in 2D inflamed hIECs

**Figure 3:**
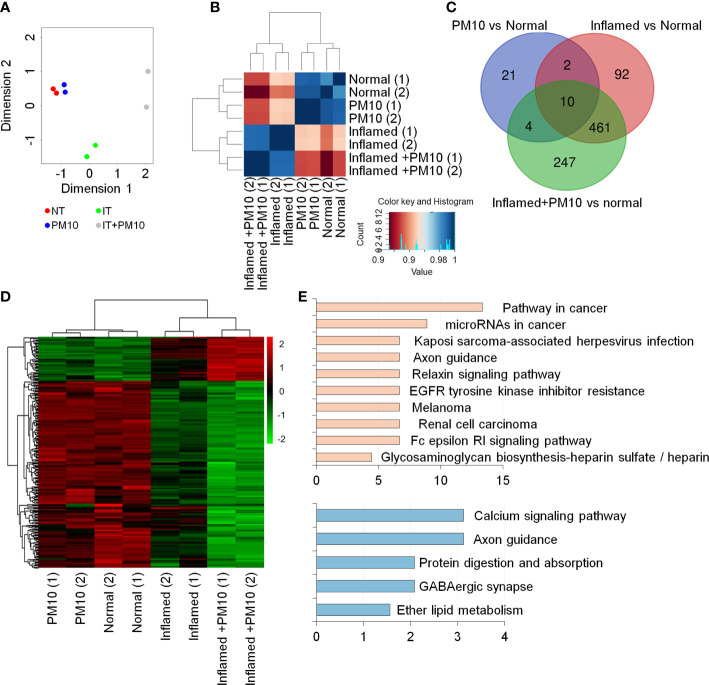
Transcriptome analysis of 2D normal and inflamed hIECs following PM10 exposure. **(A)** PCA of differentially expressed genes (DEG, fold change cut-off 2) from RNA-seq analysis compared to normal hIECs, normal hIECs exposed to PM10, inflamed hIECs, and inflamed hIECs exposed to PM10. **(B)** Spearman’s correlation analysis. Red and blue indicate the highest and lowest level of similarity, respectively. **(C)** Venn diagram of each groups using RNA-seq results for DEG. The numbers of DEG are indicated. **(D)** Heatmap with hierarchical clustering analysis for PM10-treated inflamed hIEC-specific genes. Green color represents down-regulated genes, whereas red color represents up-regulated genes compared with normal hIEC control. **(E)** The KEGG pathway enrichment analysis for PM10-treated inflamed hIECs-specific genes. The bars represent the count percentage of genes.

To examine changes in gene expression signatures in the inflamed hIEC model and the effects of PM10, RNA-seq analyses were performed on normal and inflamed hIECs with or without PM10 treatment. RNA-seq-based principal component analysis (PCA) and Spearman’s correlation analysis showed that normal and inflamed hIEC samples clustered distinctly with or without PM10 treatment (**Figures 3A, B**). Using Venn diagram analysis and hierarchical clustering, we characterized the specific response of inflamed hIEC. Among 461 inflammation-specific genes, 206 genes were upregulated and 255 genes were downregulated in inflamed hIECs regardless of PM10 treatment (**Figure 3C**). As a result of KEGG pathway enrichment analysis, various inflammatory response-related pathways, including cytokine-cytokine receptor interactions, TNF signaling, NF-kappa B signaling, and IL-17 signaling pathways were enriched. In addition inflamed hIECs displayed a decrease in ion transport regulation (**Supplementary Figure 3**). After analyzing PM10-treated inflamed hIECs for specific gene alterations, we identified 247 genes, including 47 upregulated genes and 200 downregulated genes (**Figures 3C, D**). Genes annotated as calcium signaling, protein digestion, and absorption pathways were mostly downregulated (**Figure 3E**). Therefore, we focused on calcium signaling, protein digestion and absorption pathways. As a result of these pathways, the GI tract performs a variety of biological functions, including nutrient uptake, hormone secretion, and protection of the mucosal barrier by the secretion of bicarbonate (29).

### 3.4 PM10 induced calcium signaling dysfunction disturbs nutrient uptake

**Figure 4:**
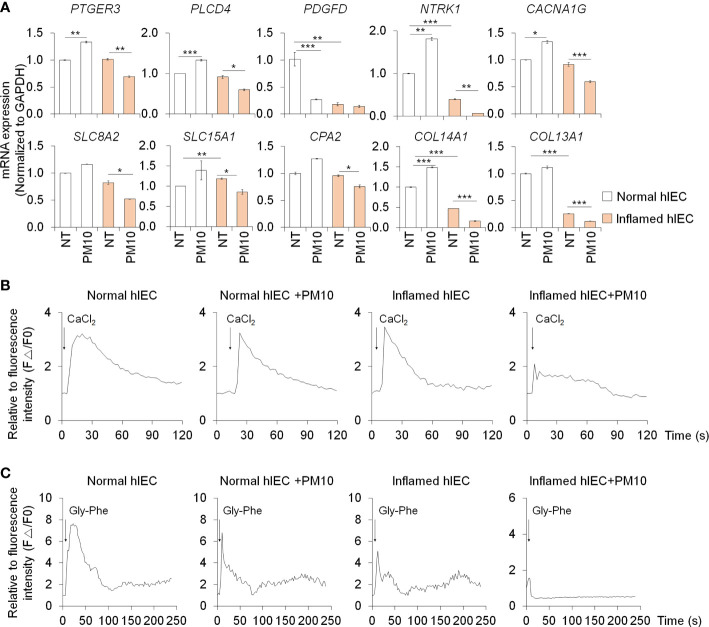
Calcium signaling dysfunction induced by PM10 exposure resulted in impaired peptide uptake. **(A)** Relative expression of calcium signaling-related genes (*PTGER3*, *PLCD4*, *PDGFD*, *NTRK1*, and *CACNA1G*) and protein absorption and digestion-related genes (*SLC8A2*, *SLC15A1*, *CPA2*, *COL13A1*, *COL14A1*). **(B)** CaCl_2_ (10 mM)-induced calcium influx in real-time measured in each group (normal hIECs, normal hIECs with PM10, inflamed hIECs, and inflamed hIECs with PM10) by using Fluo-4 AM calcium indicator. **(C)** Dipeptide (Gly-Phe)-induced calcium response in real-time was measured in each group. Quantification graph of Fluo-4 AM analysis in each group (normal hIECs, normal hIECs with PM10, inflamed hIECs, and inflamed hIECs with PM10). Scale bar = 200 μm. Data are means ± SEM (n = 3). *P < 0.05, **P < 0.01, and ***P < 0.001 by one-way ANOVA test.

The expression of calcium signaling and protein absorption pathway-related genes was further confirmed by qPCR analysis to verify the RNA-seq results. Upon treatment with PM10, the expression of calcium signaling pathway-related genes (*PTGER3*, *PLCD4*, *PDGFD*, *NTRK1*, and *CACNA1G*) and protein absorption and digestion pathway-related genes (*SLC8A2*, *SLC15A1*, *CPA2*, *COL13A1*, and *COL14A1*) decreased significantly in inflamed hIECs (**Figure 4A**). To investigate the calcium-related functionality of hIECs after exposure to PM10, we performed a calcium detection analysis using Fluo-4 AM, a well-known indicator for monitoring calcium dynamics. CaCl_2_ and Gly-Phe dipeptide were used to stimulate calcium influx and protein absorption, respectively. Following PM10 treatment, CaCl_2_ and Gly-Phe-induced calcium release significantly decreased in inflamed hIECs (**Figures 4B, C**). These results suggested that upon exposure to PM10, inflamed hIECs were more prone to abnormal calcium signaling followed by inhibition of peptide uptake. This ultimately impairs the proper functionality of intestinal epithelial cells.

### 3.5 Development of *in vitro* 3D inflamed intestinal epithelium

**Figure 5:**
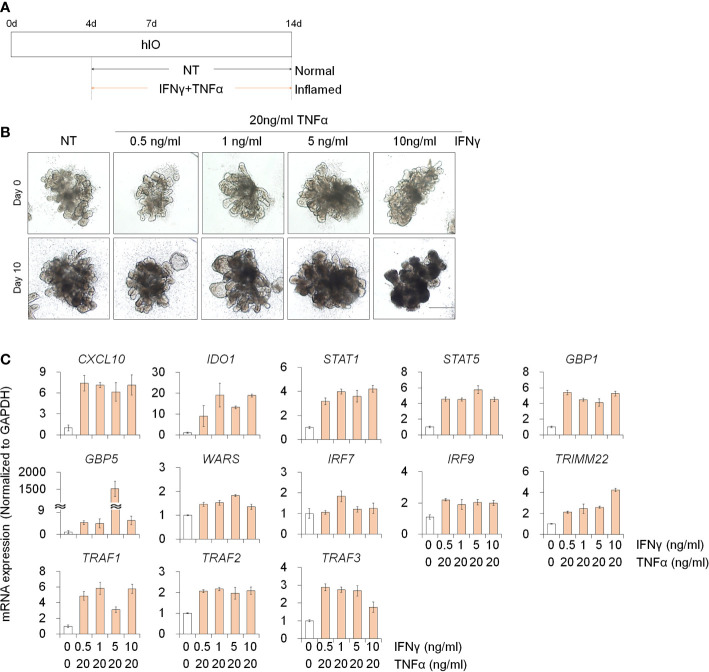
Development of 3D inflamed hIOs. **(A)** Schematic diagram of 3D inflamed hIO generation. **(B)** Morphological analysis of 3D normal and inflamed hIOs after IFNγ and TNFα co-treatment. Scale bar = 500 μm. **(C)** Relative expression of IFNγ and TNFα response genes after IFNγ and TNFα co-treatment. Data are means ± SD (n = 3). *P < 0.05, **P < 0.01, and ***P < 0.001 by two-tailed t test.

To investigate the deleterious effects of PM10 on 3D inflamed intestinal epithelial cells, we differentiated 3D hIOs and optimized the 3D hIO-based *in vitro* inflammation model induced by co-treatment with pro-inflammatory cytokines (**Figure 5A**). Intestinal cell type-specific markers were expressed in 3D hIOs (**Supplementary Figures 1C, D**). 0.1–10 ng/ml IFNγ or 2 –100 ng/ml of TNFαs were used to generate 3D inflamed hIOs (**Supplementary Figures 4A, B**). The genes that are involved in the IFNγ response (*CXCL10*, *IDO1*, *STAT1*, *STAT5*, *GBP1*, *GBP5*, *IRF7*, *IRF9*, *TRMM2*, and *WARS*) depend on the IFNγ dose (**Supplementary Figure 4C**). The 10 ng/ml IFNγ treated 3D hIOs underwent cell death (**Supplementary Figures 4B, C**). The expression of TNFα-response genes (*TRAF1*, *TRAF2*, and *TRAF3*) increased similarly in the 20–100 ng/ml TNFα treatment group; thus, we chose the lowest concentration of 20 ng/ml TNFα (**Supplementary Figures 4B, C**). We tested co-treatment with various concentrations of IFNγ and 20 ng/ml TNFα for 10 days. In each group, damaged cell morphology and upregulated expression of inflammatory response genes were observed (**Figures 5B, C**). Cell death was observed in the group treated with the highest concentration of IFNγ (10 ng/ml). Thus, an IFNγ concentration of 5 ng/ml was selected for combination treatment (**Figure 5B**).

**Figure 6:**
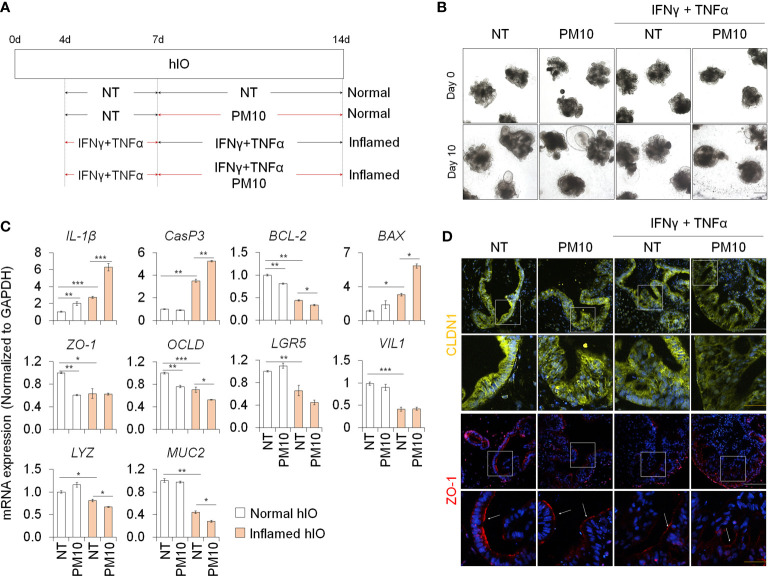
Phenotypic analysis in 3D inflamed hIOs following PM10 exposure. **(A)** Schemetic diagram of PM10 treatment in 3D normal and inflamed hIOs. **(B)** Morphological analysis of 3D normal and inflamed hIOs with or without PM10 (100 μg/ml). Scale bar = 200 μm. **(C)** Relative expression of inflammation, apoptosis, tight junction, and intestinal marker genes. Data are means ± SD (n = 3). *P < 0.05, **P < 0.01, and ***P < 0.001 by two-tailed t test. **(D)** Representative images of tight junction protein (CLDN1) in each groups. White scale bar=100μm. Yellow scale bar=50μm.

**Figure 7:**
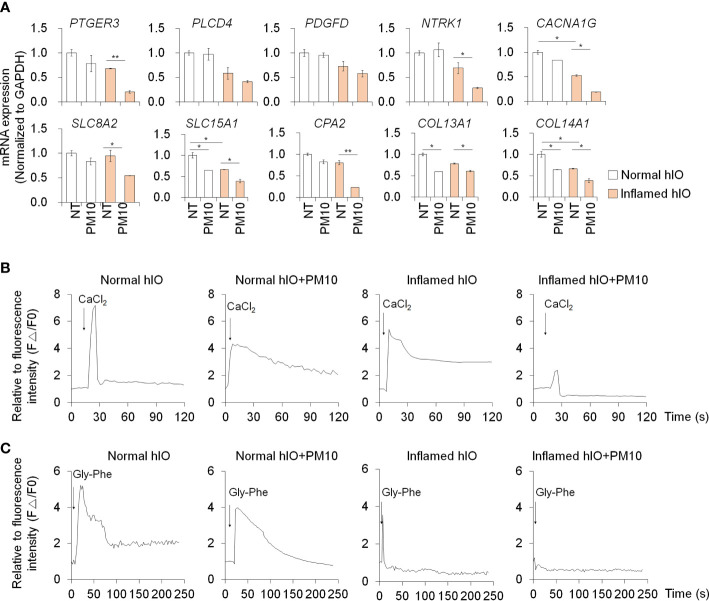
Phenotypic validation in 3D inflamed hIOs following PM10 exposure. **(A)** Relative expression analysis of calcium signaling-related genes (*PTGER3*, *PLCD4*, *PDGFD*, *NTRK1*, and *CACNA1G*) and protein absorption and digestion-related genes (*SLC8A2*, *SLC15A1*, *CPA2*, *COL13A1*, *COL14A1*). **(B)** CaCl_2_ (10 mM)-induced calcium influx in real-time measured in each group (normal hIOs, normal hIOs with PM10, inflamed hIOs, and inflamed hIOs with PM10) by using Fluo-4 AM calcium indicator. **(C)** Dipeptide (Gly-Phe)-induced calcium response in real-time measured in each group (normal hIOs, normal hIOs with PM10, inflamed hIOs, and inflamed hIOs with PM10) by using Fluo-4 AM calcium indicator. Data are means ± SD (n = 3). *P < 0.05, **P < 0.01, and ***P < 0.001 by one-way ANOVA test.

We examined the deleterious effects of PM10 exposure on control hIOs and inflamed 3D hIOs induced by co-treatment with 5 ng/ml IFNγ and 20 ng/ml TNFα (**Figure 6A**). Damaged 3D hIOs were observed in inflamed hIOs following exposure to 100 μg/ml of PM10 (**Figure 6B**). This is consistent with 2D hIECs, PM10-treated 3D inflamed hIOs increased the expression of *IL-1β* and *CasP3* and decreased the expression of *BCL-2* and intestinal specific markers (*CDX2*, *LGR5*, *VIL1*, *LYZ*, and *MUC2*) (**Figure 6C**). As a consequence of PM10 exposure, the expression of tight junction-related genes (*ZO-1* and *OCLD*) was downregulated (**Figure 6C**), and their corresponding proteins were mislocalized in 3D inflamed hIOs (**Figure 6D**). We investigated whether 100 μg/ml PM10 induced functional alterations in normal and inflamed 3D hIOs. When PM10 was applied to inflamed 3D hIOs, the expression of calcium signaling and protein absorption-associated genes were decreased (**Figure 7A**). Accordingly, PM10 treatment was less effective for normal hIOs than for inflamed ones (**Figure 7A**). Furthermore, fluo-4 AM dye intensity was significantly reduced in 3D inflamed hIOs treated with PM10 compared to other groups upon treatment with CaCl_2_ and Gly-Phe (**Figures 7B, C**). As demonstrated by these findings, PM10 exposure disrupted small intestinal functions in 3D hIOs and peptide uptake, which is consistent with the results obtained in 2D hIECs.

## 4 Discussion

Specifically, this study examines 1) the deleterious effects of PM10 exposure in 2D and 3D inflamed intestinal models *in vitro*; 2) gene expression related to calcium signaling and protein digestion and absorption pathways is dysregulated in 2D inflamed hIECs after PM10 exposure and in 3D inflamed hIOs following chronic PM10 exposure. According to these findings, PM10 might have a more deleterious effect on patients with underlying medical conditions such as inflamed intestines.

Dextran sodium sulfate-induced colitis model and IL-10-deficient mouse model are well-known conventional *in vivo* inflamed-epithelium model systems (30, 31). These model systems can be used to observe phenotypes such as immune response, crypt loss, goblet cell loss, intestinal disintegration and suggest a practical approach to study the systemic effects between the intestinal epithelial cells and immune cells. Although the immune cells are absent in *in vitro* 2D hIECs and 3D hIOs, pro-inflammatory cytokine treatment can mimic the inflamed intestinal epithelium and serve as a human intestinal epithelial model. INFγ and TNFα are well-known fundamental causes and biological therapeutic targets for IBD (32). Consistent with our data (**Supplementary Figure 2**, **Figures 2**, **5**, **6**), previous reports also showed that co-treatment with INFγ and TNFα revealed synergistic cytotoxic effects, including cell death, abnormal gut permeability, and intestinal functionality (33–35). These results correlated with the *in vivo* state. In IFNγ- and TNFα-treated inflamed epithelial cells, we observed an increase in inflammation response marker (*IL-1β*), reduced stem cell (*LGR5*) and goblet cell (*MUC2*) marker expression, and tight junction-related gene expression (*ZO-1* and *OCLD*) (**Figures 2**, **6**). These results correlated with *in vivo* phenotypes such as inflammation, crypt loss, goblet cell loss, and intestinal disintegration (31, 36, 37). Moreover, similar to *in vivo* study, PM10 exposure led to an increase in inflammatory response and intestinal disintegration in inflamed 2D hIECs and 3D hIOs (14, 38).

Previous studies have addressed environmental factors that augment inflammatory responses in INFγ and TNFα conditions (21). IFNγ and TNFα, believed to be important mediators of compromised barrier in IBD, have been shown to alter tight junction activity and to induce apoptosis in intestinal epithelial cells (39, 40). The molecular mechanisms of IFNγ and TNFα co-treatment have previously been well-identified in some signaling pathways such as JAK/STAT1/IRF1 and NF-κB/STAT3, and are reported to be downstream regulators which cause inflammatory diseases (41). For these reasons, many researchers have applied the co-treatment of IFNγ and TNFα to induce the inflammatory phenotypes and decreased homeostasis of diverse tissues and cells both *in vivo* and *in vitro* (42, 43). Our 2D and 3D models showed results similar to those reported by previous studies that involved assessing gene expression responses to IFNγ and TNFα treatment in *in vitro* IBD models and *in vivo* IBD patients (**Figures 1**, **5**, **Supplementary Figure 4**).

In a mouse model of exposure to PM inhalation, the diversity of small intestine-, colon-, and feces-derived microbiome was changed and could be postulated to be associated with gastrointestinal disease linked to PM (44). Previous studies have shown that mice directly administered PM10 orally showed greater intestinal permeability than mice treated with PBS (14, 19). There is, however, a lack of understanding of the molecular mechanisms behind the reduced functionality of the intestine, particularly in the human intestinal epithelium. Furthermore, long-term exposure to PM10 in IL-10^-/-^ mice resulted in increased histological severity and immune cell infiltration compared to that in wild-type (WT) mice (14). As demonstrated in these reports, PM10 exposure affects the additive pathology that underlies the inflammatory response. It has been shown that PM10-associated intestinal dysfunction can occur both in normal and IBD models *in vitro*, despite several *in vitro* phenotype analysis studies on IBD phenotypes. However, the precise pathophysiological mechanisms behind PM10-associated intestinal dysfunction remain unidentified. We found that PM10, as a possible exacerbation factor of IBD, enhanced inflammation, apoptosis, intestinal permeability, and intestinal functionality (**Figures 1**, **2**, **5**, **6**). Recently, experimental evidence revealed that immune responses in the pulmonary and intestinal mucosae are closely interrelated, and that gut-lung axis can control pathophysiological processes, and responses to PM exposure (12). Therefore, further studies involving advanced multi-organ models using intestinal and lung organoids are required.

Calcium homeostasis is regulated by various biological activities such as oxidative stress, inflammation, and nutritional stress (45). PM10 treatment increases the oxidative stress which leads to increased intestinal permeability and apoptosis in Caco-2 cell lines (19). In contrast, our normal 2D human intestinal epithelial models cannot observe PM10-induced intestinal damages and oxidative stress (**Supplementary Figure 5**). However, in inflamed groups, oxidative stress was observed using superoxide indicator (**Supplementary Figure 5**). The cooperation between pro-inflammatory cytokine and PM10 enhances superoxide production which is followed by calcium dysregulation and intestinal failure. Calcium signaling is implicated in the regulation of bicarbonate transporting proteins (CFTR, CaCC, Cl-/HCO3- exchangers), gut-derived hormone secretion (GLP-1), and nutrient sensing (PEPT1, Amino acid transporter, GLUT2/5, SGLT) in the small intestine (29, 46, 47). KEGG pathway analysis of the inflamed hIECs treated with PM10 exhibited dysfunction of calcium signaling as well as protein digestion and absorption pathways (**Figure 3**), which was confirmed by qPCR (**Figure 4**). PEPT1, a proton-coupled small peptide transporter, can absorb di-/tripeptides derived from food digestion. Chemically synthesized dipeptide Gly-Phe modulates PEPT1 promoter activity, which was observed in the human plasma after a protein meal and taken up by the PEPT1-mediated transport system in Caco-2 (48–50). Upon dipeptide influx, calcium-sensing receptor (CaSR) activation leads to stimulation of phospholipase C (PLC)-IP3 signaling and calcium release from the endoplasmic reticulum (ER) (51). By using the calcium indicators Fluo-4 AM and Gly-Phe, we were able to observe dysregulation of the calcium signaling pathway followed by protein absorption in 2D hIECs and 3D hIOs under inflammatory conditions and exposure to PM10. In **Figures 4**, **6**, inflamed 2D hIECs and 3D hIOs treated with PM10 displayed impaired expression of calcium signaling pathway components, leading to reduced protein uptake. Calcium signaling plays an important role in several intestinal functions, including nutrient uptake, hormone secretion, and bicarbonate transport, and maintains intestinal integrity. The intestinal tight junction barrier relies on intracellular calcium levels and calcium signaling-related proteins (52–54). Extracellular calcium depletion and osmotic stress-induced-calcium channel malfunction induce the disruption of tight junction in Caco-2 cell lines (53, 55). When the tight junction breaks due to laser injury or naturally occurring leaks, temporal calcium flashes at the damage site are observed, which increase Rho flares (short-lived activated states) resulting in reinforcement and contraction of local ZO-1 and repair of the leaked barrier (56). However, in the inflamed intestine, PM10 treatment causes a disruption of calcium homeostasis which blocks tight junction repair mechanisms. According to these results, PM10 exposure could lead to more severe intestinal dysfunction in 2D and 3D inflamed cell models compared to normal controls. Therefore, further investigations are required in order to demonstrate the therapeutic or preventive effects of modulating calcium signaling pathways in inflamed intestinal epithelial models under PM10 exposure conditions. It is possible that CaSR stimulation induces an increase in apical Cl^-^/HCO_3_^-^ exchange, anti-inflammatory effects, and regulation of hormone secretion in the GI tract, which suggests a recovery of intestinal function (57, 58).

In conclusion, we have successfully established *in vitro* inflamed intestinal epithelium models by cytokine combination using 2D hIECs and 3D hIOs. Furthermore, PM10 acts as an exacerbation factor in an inflamed milieu. The results of our experiments suggest that PM10 exposure can lead to dysregulation of calcium signaling-mediated protein absorption in 2D and 3D inflamed intestinal models. IBD patients exposed to PM10 may benefit from this as a potential therapeutic or preventive target.

## Data availability statement

The original contributions presented in the study are publicly available. This data can be found here: https://www.ncbi.nlm.nih.gov/geo/query/acc.cgi?acc=GSE227514.

## Ethics statement

The hESCs and the animal experiment were approved by the Public Institutional Review Board designated by the Ministry of Health and Welfare (No. P01-201409-ES-01) and the Institutional Animal Care and Use Committee of the Korea Research Institute of Bioscience and Biotechnology (No: KRIBB14 AEC-21245), respectively.

## Author contributions

M-YS and YS contributed to the study concept and design; YS and NS performed the 2D hIEC and 3D hIO experiments, respectively; M-YS, D-SK, S-JL, M-SL, Y-JP, AB, and WY contributed to the analysis and interpretation of the data; M-YS and YS wrote the manuscript. All authors contributed to the article and approved the submitted version.
