# Supplementary material for: Particulate matter 10 exposure affects intestinal functionality in both inflamed 2D intestinal epithelial cell and 3D intestinal organoid models
Source: Front Immunol. 2023 Jun 26;14:1168064. doi: 10.3389/fimmu.2023.1168064 (PMC10331606; doi:10.3389/fimmu.2023.1168064)
Supplement: Supplementary file 1 [file DataSheet_1.pdf]

Supplementary Material

Particulate matter 10 exposure affects intestinal functionality in both inflamed 2D intestinal epithelial cell and 3D intestinal organoid models

Ye Seul Son[1,†], Naeun Son[1,2,†], Won Dong Yu[1,2], Aruem Baek[1], Young-Jun Park[3], Moo-Seung Lee[3], Seon-Jin Lee[3], Dae-Soo Kim[4,*], and Mi-Young Son[1,2,*]

[*]Correspondence: Mi-Young Son (myson@kribb.re.kr) and Dae-Soo Kim (E-mail: kds2465@kribb.re.kr)

Supplementary Figure 1

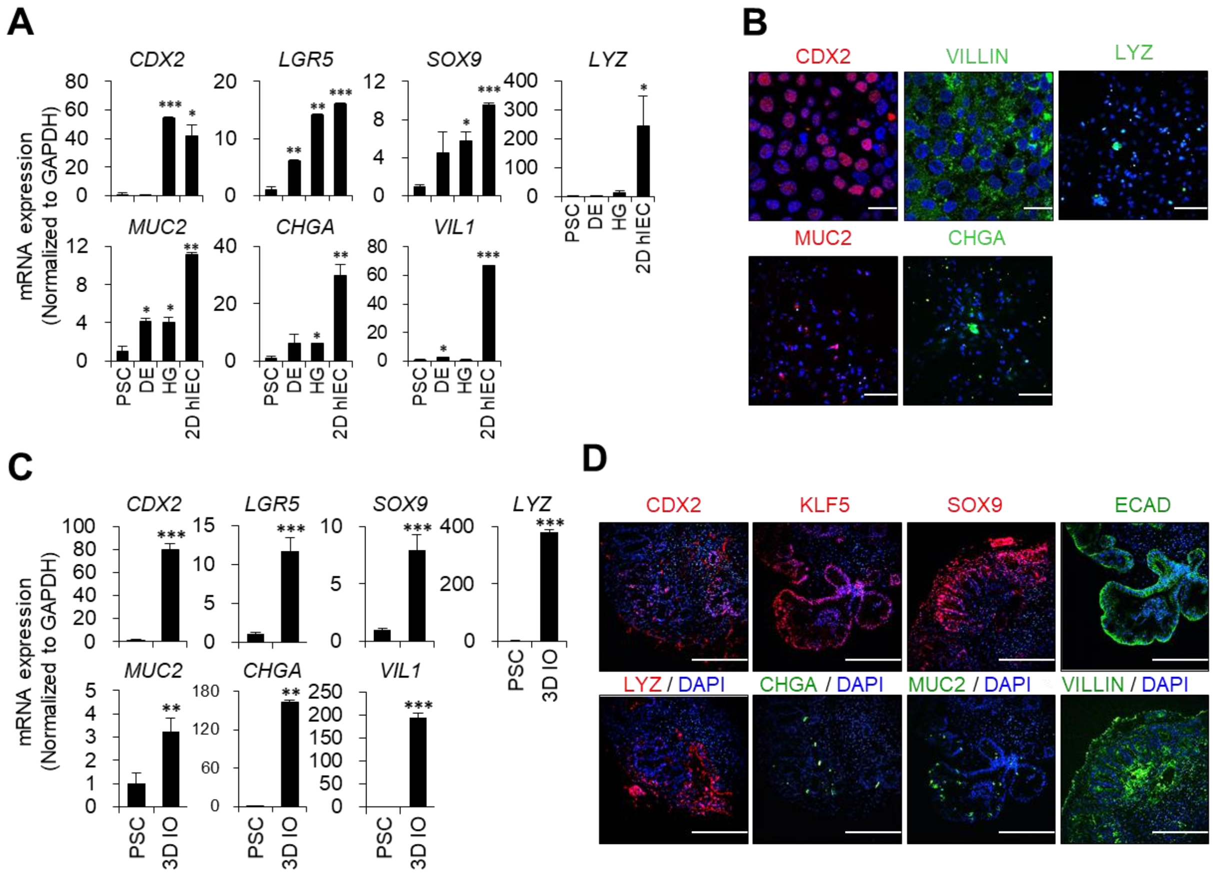

Supplementary Figure 1 Differentiation and characterization of hPSC-derived 2D hIECs and 3D hIOs. **(A) and (C)** mRNA expression of intestinal-specific markers (*CDX2*, *LGR5*, *SOX9*, *LYZ*, *MUC2*, *CHGA*, and *VIL1*). Data are presented as the mean ± SD (n = 3). *$P < 0.05$, ** $P < 0.01$, and *** $P < 0.001$ by two-tailed t test. **(B) and (D)** Representative images of intestine-specific markers, including progenitor markers (CDX2, KLF5, and SOX9), epithelial cells (ECAD), Paneth cells (LYZ), goblet cells (MUC2), enterocytes (VIL), and endocrine cells (CHGA). White scale bar: 100 µm. Yellow scale bar: 200 µm.

Supplementary Figure 2

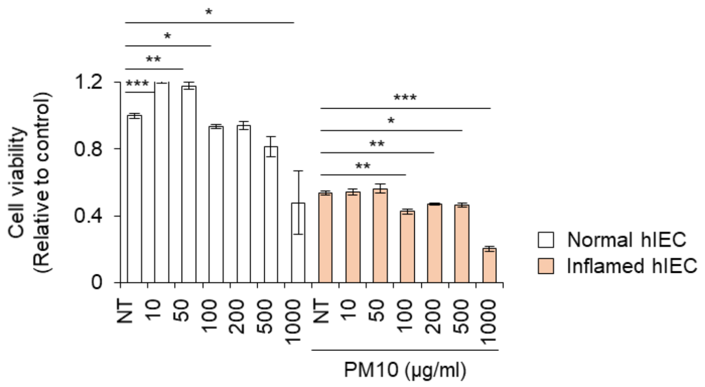

Supplementary Figure 2 Cell viability assay in 2D normal and inflamed hIECs treated with PM10. Data are presented as the mean ± SD (n = 3). *P < 0.05, ** P < 0.01, and *** P < 0.001 by two-tailed t test.

Supplementary Figure 3

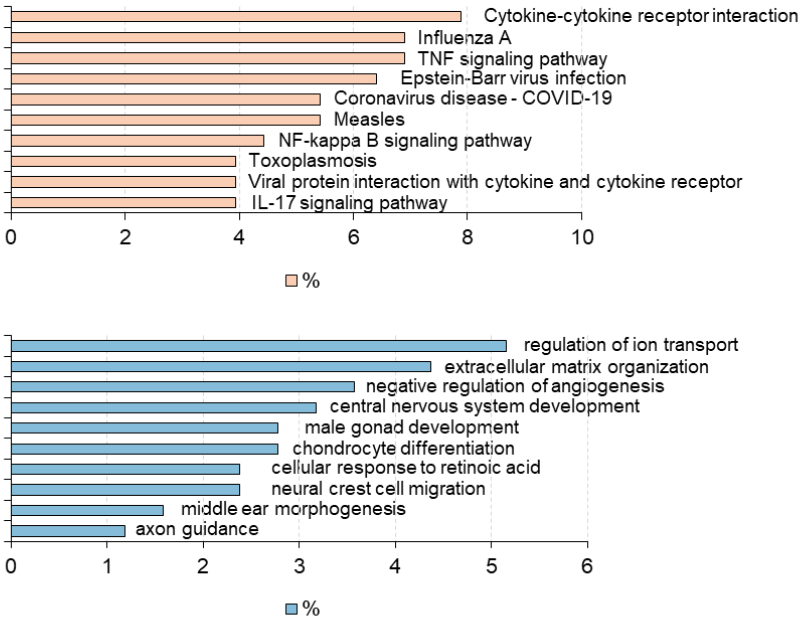

Supplementary Figure 3 KEGG pathway enrichment analysis of up- and down-regulated genes in 2D inflamed hIECs compared with normal hIECs.

Supplementary Figure 4

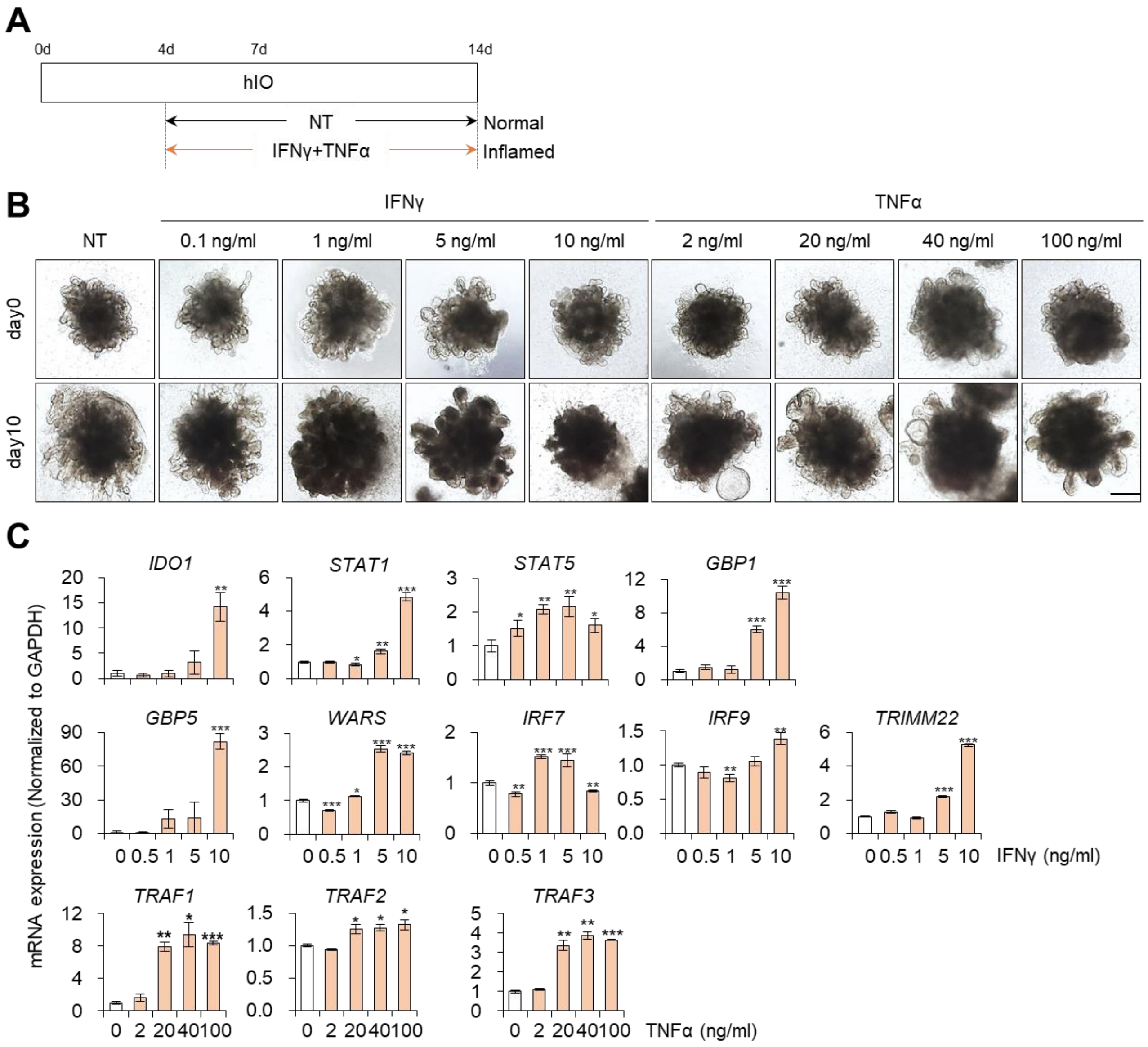

Supplementary Figure 4 Cellular responses to pro-inflammatory cytokines in 3D hIOs. **(A)** Schematic diagram of IFNγ and TNFα treatment in 3D hIOs. **(B)** Morphological analysis of 3D hIOs treated with cytokines. Scale bar = 500 μm. **(C)** Relative expression of IFN-γ (*CXCL10*, *IDO1*, *STAT1*, *STAT5*, *GBP1*, *GBP5*, *IRF7*, *IRF9*, *TRIMM22*, and *WARS*) and TNF-α (*TRAF1*, *TRAF2*, and *TRAF3*) response genes in hIOs after IFN-γ and TNF-α treatment. Data are presented as the mean ± SD (n = 3). **P* < 0.05, ** *P* < 0.01, and *** *P* < 0.001 by two-tailed t test.

Supplementary Figure 5

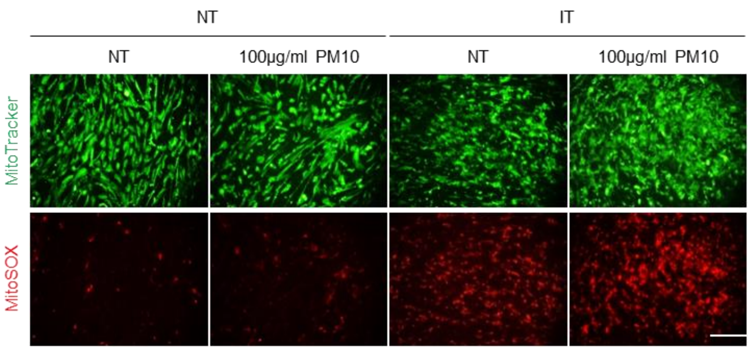

Supplementary Figure 5 Oxidative stress analysis in 2D normal and inflamed hIECs following PM10 exposure. Representative images of MitoTracker Green (Mitochondrial contents) and MitoSOX (mitochondrial superoxide). Scale bar, 200 μm.

Supplementary Table 1. List of the primers used in this study.

Gene	Primer (Forward)	Primer (Reverse)
GAPDH	GAA GGT GAA GGT CGG AGT C	GAA GAT GGT GAT GGG ATT TC
CDX2	CTG GAG CTG GAG AAG GAG TTT C	ATT TTA ACC TGC CTC TCA GAG AGC
LGR5	TGC TCT TCA CCA ACT GCA TC	CTC AGG CTC ACC AGA TCC TC
SOX9	GGA GAG CGA GGA GGA CAA GTT C	TTG AAG ATG GCG TTG GGG G
LYZ	AAA ACC CCA GGA GCA GTT AAT	CAA CCC TCT TTG CAC AAG CT
MUC2	TGT AGG CAT CGC TCT TCT CA	GAC ACC ATC TAC CTC ACC CG
CHGA	TGA CCT CAA CGA TGC ATT TC	CTG TCC TGG CTC TTC TGC TC
VIL1	AGC CAG ATC ACT GCT GAG GT	TGG ACA GGT GTT CCT CCT TC
CXCL10	TGG CAT TCA AGG AGT ACC TCT C	CGT GGA CAA AAT TGG CTT GC
IDO1	ACA CTT TGC TAA AGG CGC TG	TGC CTT TCC AGC CAG ACA AA
STAT1	ATG GCA GTC TGG CGG CTG AAT T	CCA AAC CAG GCT GGC ACA ATT G
STAT5	GTT CAG TGT TGG CAG CAA TGA GC	AGC ACA GTA GCC GTG GCA TTG T
GBP1	AAA CTT CAG GAA CAG GAG CAA C	GGT ACA TGC CTT TCG TCG TCT
GBP5	CCC AAC TTG AAA CAC TGC CTG	GCA CCA GGT TCT TTA GAC GAG A
IRF7	CCA CGC TAT ACC ATC TAC CTG G	GCT GCT ATC CAG GGA AGA CAC A
IRF9	CCA CCG AAG TTC CAG GTA ACA C	AGT CTG CTC CAG CAA GTA TCG G
TRIMM22	ACT ACT GGG TGG ACG TGA TG	GCC GAA GAC ACC AAA AGC AG
WARS	CGA CTG CAT TGG GAA GAT CAG	ATG GCA CAT GGG ATA AGG CAC
TRAF1	CGA TGG CAC TTT CCT GTG GAA G	TAC AGC CGC AGG CAC AAC TTG T
TRAF2	GAG CAG AAG GTC TTG GAG ATG G	GCA GAC ACA TCT TGT AGC CGT AC
TRAF3	ACA AGT GCA GCG TCC AGA CTC T	GCC TTG ATC TGC TGG TTT GTC C
IL-1β	GGG CCT CAA GGA AAA GAA TC	TTC TGC TTG AGA GGT GCT GA
CasP3	TGG AAT TGA TGC GTG ATG TT	GGC AGG CCT GAA TAA TGA AA
BCL-2	TTT TAG GAG ACC GAA GTC CG	AGC CAA CGT GCC ATG TGC TA
BAX	CCT GTG CAC CAA GGT GCC GGA ACT	CCA CCC TGG TCT TGG ATC CAG CCC
ZO-1	CCC GAC CAT TTG AAC GCA AG	ATG CCC ATG AAC TCA GCA CG
OCLD	CAT TGC CAT CTT TGC CTG TG	AGC CAT AAC CAT AGC CAT AGC
PTGER3	CCT TCA AGG TTC TGT GCT CAG C	CAT CAG CTT AGC TGG ACA CTG C
PLCD4	GAA CCT GTC GTT TAC CAC GGA C	CAG GGA CAA GAT GAC TGG GTA G
PDGFD	GCG GCT TCA CTC TCA GGA GAA T	CTT GTG TCC ACA CCA TCG TCC T
NTRK1	CAC TAA CAG CAC ATC TGG AGA CC	TGA GCA CAA GGA GCA GCG TAG A
CACNA1G	TTC ACC GCA GTC TTT CTG GCT G	TGA CGG AGA TGA GCA CCA ACA G
SLC8A2	GGA GCA TCT TCG CCT ATG TCT G	ATC CAG GCG AAT ACC ACG CAC A
SLC15A1	AAG TCG GTG CTT CAG GCA GGA T	ACA CAG ACG ACC AGA AGC AAC G
CAP2	CCA TCA CTT CCA TTC TGG ACG C	CAC CAA CAC AGA GGC TTC CAG A
COL14A1	CAC AAA CCT CCT CAG CGG AAT G	GGC TTG GAG ATT GGT AAC ACC C
COL13A1	TGG AGA ACA GGG ACC AGA TGG C	GAT CTC CTG GAG AGC CTC ATT G

Supplementary Table 2. List of antibodies used in this study.

Antibodies	Company	Catalog No.	Dilution
anti-ZO-1	Thermo Scientific	61-7300	1:40
anti-Claudin1	Abcam	Ab15098	1:100
anti-CDX2	Biogenex	AM392-5M	1:100
anti-VILLIN	SantaCruz	SC-7672	1:50
anti-LYZ	Abcam	Ab76784	1:200
anti-MUC2	SantaCruz	SC-7314	1:50
anti-CHGA	Thermo	MA5-14536	1:200
anti-KLF5	Abcam	ab137676	1:100
anti-SOX9	SantaCruz	SC-20095	1:50
